# Synthetic rabbit-human antibody conjugate as a control in immunoassays for immunoglobulin M specific to hepatitis E virus

**DOI:** 10.1186/1743-422X-7-101

**Published:** 2010-05-20

**Authors:** Kuo Zhang, Lunan Wang, Min Liu, Rui Zhang, Jinming Li

**Affiliations:** 1 National Center for Clinical Laboratories, Beijing Hospital, Beijing, PR China

## Abstract

**Background:**

In assays for anti-hepatitis E virus (HEV) immunoglobulin M (IgM), large volumes of the patient's sera cannot be easily obtained for use as a positive control. In this study, we investigated an alternative chemical method in which rabbit anti-HEV IgG was conjugated with human IgM and was used as a positive control in the anti-HEV IgM assay. Rabbit anti-HEV IgG was isolated from immune sera by chromatography on protein A-Sepharose and was conjugated with human IgM by using 1-ethyl-3-(3-dimethylaminopropyl)carbodiimide (EDC) as a crosslinker.

**Results:**

The specific anti-HEV IgG antibody titer was 100,000 times that of the negative control, i.e., prebleed rabbit serum. The results of anti-HEV IgM enzyme-linked immunosobent assay showed that the antibody conjugate was similar to anti-HEV IgM antibodies produced in humans. The results of a stability experiment showed that the antibody conjugate was stable for use in external quality assessment or internal quality control trials.

**Conclusions:**

We concluded that the chemically conjugated rabbit-human antibody could be used instead of the traditional serum control as a positive control in the anti-HEV IgM assay.

## Background

Hepatitis E, the major form of enterically transmitted non-A, non-B hepatitis, is caused by hepatitis E virus (HEV) [1-3]. Although an efficient cell-culture system for HEV has been developed and evaluated, this system cannot be easily employed in current clinical practice because several weeks are required to culture the virus [4]. Viremia is thought to be present in the serum only during the acute phase of illness, and it subsides soon after the onset of the icteric phase as HEV antibodies developing [5-7]. The diagnosis of HEV infection is mainly dependent on serological examinations [7,8]. The immunoglobulin M (IgM) antibody-capture enzyme-linked immunosorbent assay (ELISA) was specifically designed to detect IgM antibodies and is a valuable marker for rapid diagnosis of acute viral infection [6,9,10].

However, in some cases, the anti-HEV IgM assay had relatively low sensitivity. The low sensitivity of this assay may be attributed to the following 3 factors [8]: delayed sampling, sequence variations among different HEV genotypes, and poor host immune response to HEV infection in some acute patients. A positive control can be included in each run to identify the analytical problems that may lead to false-negative results. Although a positive control will not increase the sensitivity of the test, it will provide information that will facilitate the identification of problems in one test and will help to improve the overall quality of HEV screening procedures.

Traditionally, a positive control is a complex of seropositive plasma or serum with known quantities of anti-HEV IgM and the negative control reagent. However, the use of plasma or serum as positive control has several significant drawbacks, including low stability, high cost [11,12], difficulty in economically viable large-scale production because the decline in the concentration of IgM antibodies against HEV is steeper than that of total Ig during the first 3 months [13-15], and potential infection with reported 51% PCR positive when both IgG and IgM was present in serum [16].

Engineered human antibodies with murine variable regions and human constant regions have been developed as an alternative positive control; these antibodies have overcome some drawbacks of the traditional seropositive control. These antibodies were successfully used as positive controls in ELISA for *Toxoplasma gondii *and scrub typhus. However, the use of hybridoma technology and recombinant DNA technology has increased the time required to prepare these antibodies. The affinity of engineered antibodies specific for 1 epitope on a single antigen is different from those of antibodies produced in humans; which also encumbered the usage in immunoassays for different antigens or different epitopes on the same antigen [11,17-19].

We investigated the feasibility of replacing the conventional positive controls with an antibody conjugate in which rabbit anti-HEV IgG was conjugated with human IgM by using 1-ethyl-3-(3-dimethylaminopropyl) carbodiimide hydrochloride (EDC) as a crosslinker [20].

## Methods

### Immunization

We injected 2 New Zealand white rabbits with the HEV antigen. Because anti-HEV assays based on open reading frame 2 (ORF2) were shown to be more sensitive than those based on ORF3 [21], we used recombinant protein NE2 (supplied by Wantai Biotechnology Company [China]) with an ORF2 immunodominant epitope as the antigen. We dissolved 10 μl of the antigen (1.817 mg/ml) in 0.9% sodium chloride. Subsequently, we emulsified 1 ml of this solution with the same volume of Freund's complete adjuvant (Sigma, Ameriaca) and administered 1 ml of the emulsion to each rabbit; the emulsion was distributed among 8 intradermal sites on the rabbit's back. Subsequently, 6 half-dose booster injections were administered with Freund's incomplete adjuvant (Sigma, Ameriaca) as above at intervals of 2 weeks, and 1 ml of blood was withdrawn from the jugular vein each time to determine the antibody titer.

### Detection of antibodies

We first performed indirect ELISA with untreated sera as a crude test to detect the HEV antibody titer. The serum samples were diluted to the following dilutions 1:100, 1:1,000, 1:10,000, and 1:100,000 by using a coating buffer (50 mmol/L sodium carbonate buffer [pH9.5]). We used 100 μl of the appropriate dilution to coat the well of the microtiter plate. After overnight incubation at 4°C, the wells were washed 5 times using a washing buffer (10 mmol/L phosphate buffered saline (PBS) [pH9.5]). Subsequently, 150 μl of a blocking buffer (20% bovine serum in PBS) was added to each well. The microtiter plates were incubated for 2 h at 37°C and then washed 5 times using the washing buffer. We added 100 μl of horseradish peroxidase (HRP)-labeled HEV-NE2 antigen (Wantai Biological Pharmacy Enterprise Co., Ltd., Beijing) to each well, and the plate was incubated at 37°C for 15 min. Further, 50 μl of substrate buffer (phosphate citric acid buffer [pH 9.5]) and 50 μl of tetramethylbenzidine (TMB) colorization buffer were added to each well, and the plate was incubated at 37°C for 15 min. Subsequently, 50 μl of stop solution (2 mol/L H_2_SO_4_) was added to each well. Optical density (OD) values were measured using an enzyme immunoassay (EIA) plate reader with 2 filters of 450 nm/620 nm.

Blood with high IgG titer was collected by carotid artery bleeding after 3 months, and the sera were stored at -20°C.

### Antibody Purification

Rabbit IgG was isolated from immune sera by protein A-Sepharose chromatography using HiTrap affinity columns (HiTrap™; Protein A HP, Amersham Bioscience). The elution buffer was 0.58% (v/v) acetic acid NaCl (0.15 mol/L) solution. Purification was performed according to the manufacturer's instructions. The purified IgG was quantified using an Eppendorf biophotometer; then, purity was evaluated by sodium dodecyl sulfate-polyacryl-amide gel electrophoresis (SDS-PAGE).

### Synthesis of antibody conjugate

Carboxyl and amine-reactive zero-length crosslinker EDC was obtained from Pierce. 2-(*N*-morpholino) ethane sulfonic acid (MES) was purchased from Sigma. Human IgM was from Chemicon International, USA.

The neutralized IgG fraction and human IgM immunoglobulin were dialyzed overnight at 4°C with stirring against conjugation buffer (0.1 mol/L MES sodium chloride, pH 4.5). We added 1 mg each of the IgG fraction and human IgM into 100 μl of conjugation buffer. The reaction mixture was incubated at room temperature for 2 h. Subsequently, the mixture was dialyzed overnight at room temperature with stirring against PBS to remove crosslinker and other by-products.

Indirect ELISA was performed with the purified rabbit anti-HEV IgG and the antibody conjugate by using the method described in the section on detection of antibodies. OD values were measured using an EIA plate reader with 2 filters of 450 nm/620 nm. Conjugation efficiency was calculated by the following equation: conjugation efficiency = (OD for the antibody conjugate/OD for the purified rabbit anti-HEV IgG) × 100%.

To determine whether the antibody conjugate was similar to human samples, the conjugate and positive control serum from patients with hepatitis E were respectively diluted in 20% newborn calf serum and PBS to dilutions of 1:10, 1:20, 1:40, 1:80, 1:160, 1:320, 1:640. Anti-HEV IgM ELISA was performed using commercial kits (Beijing Wantai Biological Pharmacy Enterprise Co. Ltd) according to the protocols provided by the manufacturer. The assay results for the conjugate were compared with those for positive control serum from the patients. The antibody conjugate was tested in duplicate in coating buffer, and the P/N values (P and N indicate OD values for the positive and negative controls, the negative control used at this instance was prebleed rabbit serum) were compared with the OD values for unconjugated rabbit anti-HEV IgG.

### Stability of the antibody conjugate

We examined the stability of the antibody conjugate. Initially, the antibody conjugate was diluted 1:20 in PBS (0.01 mol/L, pH 7.4) containing 1% bovine serum albumin (BSA) and 10 mM histidine. Further, we incubated 500 μl of stock solution, in duplicate, at -20°C, 4°C, 37°C, and room temperature for 0, 1, 2, 4, and 8 weeks. Samples were removed at each time point and the antibody titer was measured by ELISA using an anti-HEV IgM Diagnostic Kit (Wantai, Beijing).

## Results

### Detection of rabbit anti-HEV antibody titer

The results of ELISA (OD values) indicated that after the sixth booster injection, the antibody titer in rabbit sera was 100,000 times that of the negative control that was prebleed rabbit serum (data not shown).

### Purity of the rabbit IgG

The purity of IgG was evaluated by SDS-PAGE analysis. The bands that showed the maximum staining were observed at 50 kDa and 25 kDa, and these bands corresponded to the molecular weights of heavy and light chains of IgG, respectively. No other minor bands were found in the sample. The sample was pure, and the efficiency of sample purification was about 76.9%. The absorbance of purified IgG was measured at 280 nm, and the concentration of IgG was about 10 mg/ml.

### Detection of the rabbit (IgG)-human (IgM) antibody conjugate

The P/N ratio of unconjugated rabbit anti-HEV IgG (2.5) was less than 198, which was P/N ratio of the rabbit (IgG)-human (IgM) antibody conjugate, thereby showing that the conjugate had been constructed. The efficiency of conjugation was (49.75 ± 1.2)% (n = 3).

**Figure 1:**
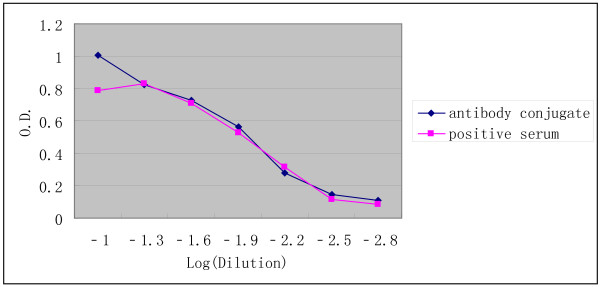
**Comparison between chimeric antibody and antibody-positive serum**. The comparison between endpoint titers of the antibody conjugate and anti-hepatitis E virus (HEV) immunoglobulin M (IgM)-positive serum in anti-HEV IgM enzyme-linked immunosorbent assay (ELISA) revealed no significant difference between the titers of positive serum-derived control and the antibody conjugate.

Figure 1 shows the dilution curves for the conjugate and the serum-derived controls in anti-HEV IgM ELISA. The endpoint titers of both the antibody conjugate and anti-HEV IgM positive serum were 1:160, which revealed that no significant difference was present between the positive serum-derived control and the antibody conjugate.

### Stability of the rabbit (IgG)-human (IgM) antibody conjugate

**Figure 2:**
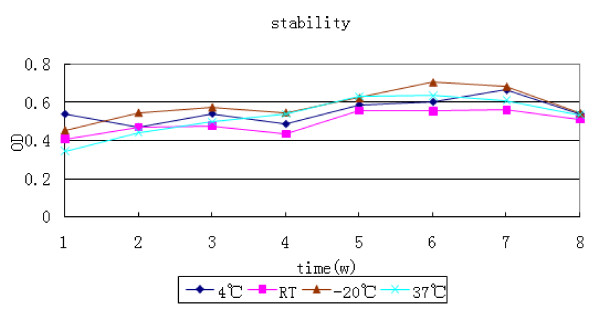
**Antibody conjugate stability analysis**. The antibody conjugates were diluted 1:20 with phosphate buffered saline (PBS) (0.01 mol/L, pH 7.4) containing 1% bovine serum albumin (BSA) and 10 mM histidine. After incubation at 20°C, 4°C, 37°C, and room temperature for 0, 1, 2, 4, and 8 weeks, samples were removed at each time point and analyzed by enzyme-linked immunosorbent assay (ELISA) using a HEV IgM Diagnostic Kit. The results showed that the antibody conjugate was stable enough to be used in external quality assessment and internal quality control trials.

The stability of the antibody conjugate was tested over a period of 8 weeks. The results obtained at each time point indicated that the antibody conjugate was stable for at least 2 months at 37°C, 4°C, room temperature, and -20°C. The results also showed that the antibody conjugate was completely stable at different temperature conditions for different periods. As shown as in Figure 2, the antibody conjugate could be delivered at room temperature and stored at -20°C or at -4°C when used in external quality assessment (EQA) or internal quality control (IQC) trials.

## Discussion

In this study, we investigated a chemical method in which rabbit anti-HEV IgG was conjugated with human IgM and used as the positive control in immunoassays for the detection of anti-HEV IgM. The synthetic rabbit-human antibody conjugate offers several advantages over the traditional methods that use engineered human antibodies or the patient's serum. First, this antibody conjugate can be produced on a large scale and it is relatively stable. Second, large volume with high titers of the antibody conjugate can be easily obtained with no risk of infection and no economic or ethical problems, and the antigen-binding behavior of these conjugates is similar to that of the clinical samples. Third, the antibody conjugate derived from polyclonal antibodies has more similarity with the human sample than engineered antibodies, because polyclonal antibodies can recognize multiple epitopes on HEV.

To improve the stability of the conjugate, it was diluted in PBS (0.01 mol/L, pH 7.4) containing 1% BSA and 10 mM histidine [22]. The antibody conjugate was stable at different temperature conditions for different periods, and therefore, it could be used in EQA or IQC trials. Further studies to determine the stability of this antibody conjugate are in progress. When the antibody conjugate is used as quality control in EQA trials, it can be delivered by mail shipped at room temperature and kept at -20°C or -4°C.

The rabbit-human antibody conjugate described in this study offers an alternative method for the preparation of a positive control that can be used in IQC and EQA trials. We have shown for the first time a chemical conjugation method to construct an anti-HEV IgM positive control. This method can be also used to construct a series of positive controls that are applicable to all immunoassays, such as ELISA, immunoblot, and immunofluorescent assay (IFA), to detect the presence of IgM molecules specific for given antigens, for example, hepatitis A virus, *T. gondii*, cytomegalovirus, herpes simplex virus, and Rubella virus.

## Conclusions

We have constructed an alternative positive control using rabbit anti-HEV IgG conjugated with human IgM for the detection of anti-HEV IgM. The conjugate offers several advantages over the traditional positive controls used in anti-HEV IgM detection

## Competing interests

The authors declare that they have no competing interests.

## Authors' contributions

JL conceived the research and wrote and edited the manuscript. KZ developed the conceptual aspects of the work, performed the experiments, and wrote the manuscript. All authors participated in data collection and read and approved the final manuscript.
